# *Staphylococcus aureus* Isolates from Goat and Sheep Milk Seem to Be Closely Related and Differ from Isolates Detected from Bovine Milk

**DOI:** 10.3389/fmicb.2016.00319

**Published:** 2016-03-14

**Authors:** Axel Merz, Roger Stephan, Sophia Johler

**Affiliations:** Institute for Food Safety and Hygiene, Vetsuisse Faculty, University of ZurichZurich, Switzerland

**Keywords:** *Staphylococcus aureus*, sheep, goat, clonality, enterotoxin genes, virulence gene profile, mastitis

## Abstract

Dairy goat and sheep farms suffer severe economic losses due to intramammary infections, with *Staphylococcus aureus* representing the main cause of clinical mastitis in small ruminants. In addition, *S. aureus* contamination of goat and sheep milk may cause staphylococcal food poisoning, as many traditional caprine and ovine milk products are not subjected to pasteurization. Data on virulence and antimicrobial resistance genes, as well as on the clonality of *S. aureus* detected in goat and sheep milk is scarce. Therefore, it was the aim of this study to determine (i) *spa* types and clonal complexes (CC) and (ii) virulence and resistance gene profiles of *S. aureus* isolated from goat and sheep milk. A total of 162 milk samples from sheep and goats presenting signs of an intramammary infection and 104 bulk milk samples were collected. While low prevalence rates of *S. aureus* was detected on single animal level, 46% of the bulk tank milk samples from small ruminants were positive for *S. aureus.* All isolates were *spa* typed and CC and virulence and resistance gene patterns were determined using a DNA microarray. Data from 49 *S. aureus* isolates was included in the statistical analysis and the construction of a SplitsTree. The analyzed isolates could be assigned to eleven CC, with the large majority of goat and sheep isolates being assigned to CC130 and CC133. The findings of this study suggest that *S. aureus* shows pronounced adaptation to small ruminants in general, but not to sheep or goats in particular. Although some common characteristics among *S. aureus* from caprine, ovine, and bovine milk samples were observed, *S. aureus* from small ruminants seem to form a distinct population. As 67% of the detected *S. aureus* strains exhibited at least one enterotoxin gene, many caprine, or ovine raw milk products may be contaminated with low levels of enterotoxigenic *S. aureus*, stressing the importance of strict maintenance of the cold chain.

## Introduction

Being one of the predominant causes of food poisoning worldwide, *Staphylococcus aureus* is of particular concern to the dairy industry (Oliver et al., 2009). Dairy sheep and goat farms also suffer severe economic losses due to staphylococcal intramammary infections, with *S. aureus* being the main cause of clinical mastitis in small ruminants (Bergonier et al., 2003). However, identification of affected animals can be challenging, as in contrast to cattle, high somatic cell counts and positive results in the California mastitis test are not necessarily reliable indicators of intramammary infections among small ruminants.

Over the last decade, the production of caprine and ovine milk in Switzerland has been increasing, with 14,000 registered small ruminant farms and a total population of approximately 490,000 heads in 2014 (Swiss Federal Statistical Office). *S. aureus* is one of the most commonly found pathogens in raw caprine and ovine milk (Marogna et al., 2012) and has been detected in over 30% of the examined raw milk of Swiss dairy goat and sheep farms (Muehlherr et al., 2003). As goat and sheep milk are often used for traditional, unpasteurized products such as raw milk cheeses, they represent a potential source of staphylococcal food poisoning (SFP).

The Centers for Disease Control estimate a total number of 240,000 SFP cases per year in the US (Scallan et al., 2011). In the EU, the number of SFP outbreaks is rising, with 386 SFP outbreaks reported in 2014 (Anonymous, 2015). SFP patients present with violent vomiting and diarrhea upon ingestion of staphylococcal enterotoxins pre-formed by *S. aureus* in food (Tranter, 1990). Many different staphylococcal enterotoxins and enterotoxin-like superantigens have been described (Dinges et al., 2000). There is evidence demonstrating emetic activity in humans for all classical enterotoxins SEA-SEE (Dinges et al., 2000) and recently also for some newly described enterotoxins (Jørgensen et al., 2005; Johler et al., 2015).

While the population structure and the genomic characteristics of *S. aureus* from bovine milk are very well described, similar data on *S. aureus* isolated from small ruminants is scarce (Scherrer et al., 2004; Concepción Porrero et al., 2012; Gharsa et al., 2012; Linage et al., 2012; Eriksson et al., 2013; Smith et al., 2014). Data on virulence and antimicrobial resistance genes, as well as on the clonality of *S. aureus* detected in goat and sheep milk is crucial to determine potential routes of transmission, to improve management strategies of affected herds, and to develop effective therapeutic interventions. Therefore, it was the aim of this study to determine clonal complexes (CC) and virulence and resistance gene profiles of *S. aureus* isolated from goat and sheep milk.

## Materials and Methods

### Bacterial Isolation and DNA Extraction

In this study, 162 milk samples of goats (*n* = 31) and sheep (*n* = 131) exhibiting one or several signs of mastitis (increased somatic cell counts, positive California mastitis test, decreased milk yield), as well as 104 raw bulk milk samples were collected from dairy farms in Switzerland (goat farms: *n* = 57; sheep farms: *n* = 47) from March to October 2015. EN ISO 6888-2 was followed for isolation of coagulase-positive staphylococci. One single colony of each different morphology exhibiting an opaque fibrin halo on rabbit plasma fibrinogen agar (Oxoid, Basel, Switzerland) was subcultured. The subcultures were grown on 5% sheep blood agar at 37°C overnight. Chromosomal DNA extraction was performed using the DNeasy Blood and Tissue Kit (Qiagen, Hilden, Germany) following the manufacturer’s instructions.

### Staphaurex Latex Agglutination Test

All *S. aureus* isolates were subjected to the Staphaurex latex agglutination test (Oxoid, Basel, Switzerland) following the manufacturer’s instructions. This assay targets microbial surface components recognizing adhesive matrix molecules (SpA, ClfA, FnBPA, and FnBPB) and frequently yields false-negative results in bovine *S. aureus* (Stutz et al., 2011; Moser et al., 2013).

### DNA Microarray, SplitsTree Analysis, and Comparison to Bovine Isolates

DNA microarray analysis was performed using Staphytype genotyping kit 2.0 (Alere, Jena, Germany) following the manufacturer’s instructions. The DNA microarray used in this study determines the presence or absence of over 300 different genes and allelic variants, and allows for assignment of CC (Monecke et al., 2008). All presumptive *S. aureus* isolates were further characterized by DNA microarray profiling, which also served as a tool for species confirmation. The DNA microarray hybridization results of isolates from goats and sheep were compared to those of isolates from an unrelated collection of 78 bovine *S. aureus* strains that were obtained in a comprehensive study investigating mastitis isolates from cows in Switzerland (Moser et al., 2013). The resistance and virulence gene profiles of the caprine, ovine, and bovine isolates were visualized using SplitsTree4^1^ as previously described (Wattinger et al., 2012).

### *spa* Typing

*spa* typing, a high resolution single-locus typing technique in *S. aureus*, was performed as previously described (Johler et al., 2011). Briefly, PCR amplicons of the polymorphic X region of the *spa* gene were purified using the GenElute PCR Purification Kit (Sigma–Aldrich, St. Louis, MO, USA), and were subsequently sequenced and assigned to *spa* types^2^.

### Inclusion Criteria

Stringent inclusion criteria were employed to avoid bias over-representation of strains isolated from both single animals and bulk milk samples of the same dairy farm: only one *S. aureus* isolate was considered for construction of the SplitsTree and statistical analysis, if the analyzed isolates exhibited the same *spa* type and ≤3 different hybridization results in the DNA microarray profiling. Two single animal isolates from sheep were therefore excluded from the study, resulting in 49 *S. aureus* isolates taken into consideration for further analyses.

### Statistical Analysis

Statistically significant differences in the distribution of virulence and resistance genes between the bovine, caprine, and ovine isolates were assessed by either Chi squared test or Fisher’s exact test (in case *n* < 5) using SPSS 23.0 (IBM Corp., Armonk, NY, USA).

## Results

A total of 162 milk samples (goats: *n* = 31; sheep: *n* = 131) of animals presenting signs of an intramammary infection and 104 bulk milk samples (goat farms: *n* = 57; sheep farms: *n* = 47) were collected. On the level of single animals, none of the goat milk samples and 2% (*n* = 3) of the sheep milk samples were positive for *S. aureus*. On the level of bulk milk samples, 60% (*n* = 34) of goat bulk milk samples and 30% (*n* = 14) of sheep bulk milk samples were positive for *S. aureus*, which equals an overall prevalence of 46% among the examined bulk milk samples of small ruminants.

**FIGURE 1:**
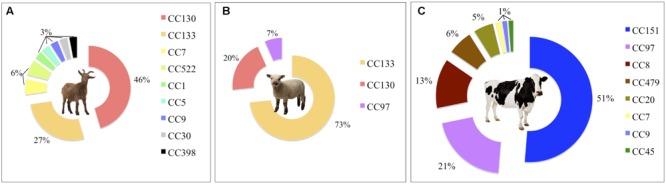
**Distribution of clonal complexes (CC) among *Staphylococcus aureus* isolated from the milk of different animal species: **(A)** goat (*n* = 34, pink), **(B)** sheep (*n* = 15, blue), **(C)** cows (*n* = 78, green)**.

**FIGURE 2:**
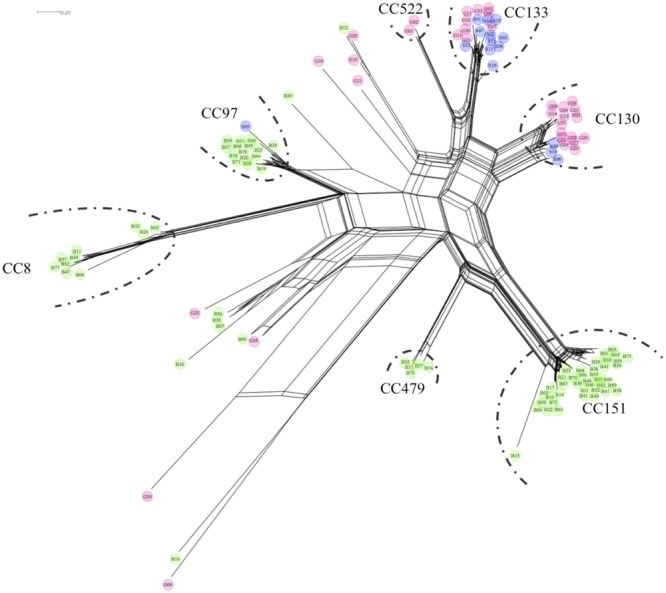
**The SplitsTree illustrates similarities between virulence and resistance gene profiles of goat (G), sheep (S), and bovine (B) *S. aureus* isolates.** The isolates grouped into clusters mainly based on assignment to CC. Isolates from small ruminants form a distinct population and were mainly found in the clusters of CC130 and CC133. Bovine isolates were predominant in clusters CC8, CC97, CC151, CC479.

*S. aureus* from small ruminants were compared to bovine mastitis isolates from the study of Moser et al. (2013), with results being presented in **Figures 1** and **2**, as well as in **Table 1**. The distribution of CC among caprine, ovine, and bovine strains is depicted in **Figure 1**, and a SplitsTree comparing gene profiles of caprine, ovine and bovine strains is shown in **Figure 2**. In general, bovine isolates and isolates from small ruminants represent distinct populations, with CC130 and CC133 exclusively associated with small ruminants. Six main SplitsTree clusters, corresponding to CC CC8, CC97, CC130, CC133, CC151, and CC479, were identified. Isolates not associated with one of the main SplitsTree clusters were assigned to CC1, CC5, CC7, CC9, CC30, CC101, and CC398.

**Table 1:** Prevalence rates of selected virulence and resistance genes detected among *Staphylococcus aureus* strains isolated from goat (G), sheep (S), and bovine (B) milk samples.

Group	Gene/Probe	Function	G (*n* = 34)	S (*n* = 15)	B (*n* = 78)
*agr*	*agrI*	Accessory gene regulator, type 1	44^∗S^	80^∗G,B^	41^∗S^
	*agrII*	Accessory gene regulator, type 2	6^∗B^	0^∗B^	59^∗G,S^
	*agrIII*	Accessory gene regulator, type 3	50^∗S,B^	20^∗G,B^	0^∗G,S^
Capsule	*cap5*	Capsule type 5	9^∗B^	7^∗B^	38^∗G^
	*cap8*	Capsule type 8	91^∗B^	93^∗B^	62^∗G^
Enterotoxins	*sea*	Enterotoxin A	3	0	10
	*sea* (320E)	Enterotoxin A, allelic variant 320E	0	0	9
	*sea* (N315)	Enterotoxin A, allelic variant N315	9	0	1
	*seb*	Enterotoxin B	0	0	0
	*sec*	Enterotoxin C	50^∗B^	67^∗B^	15^∗G^
	*sed*	Enterotoxin D	0^∗B^	0	13^∗G^
	*see*	Enterotoxin E	0	0	0
	*seg*	Enterotoxin G	9^∗B^	0^∗B^	65^∗G,S^
	*sei*	Enterotoxin I	9^∗B^	0^∗B^	65^∗G,S^
	*sek*	Enterotoxin K	0	0	0
	*sel*	Enterotoxin L	50^∗B^	67^∗B^	15^∗G,S^
	*selm*	Enterotoxin-like protein M	9^∗B^	0^∗B^	64^∗G,S^
	*seln*	Enterotoxin-like protein N	9^∗B^	0^∗B^	65^∗G,S^
	*selo*	Enterotoxin-like protein O	9^∗B^	0^∗B^	65^∗G,S^
	*seq*	Enterotoxin Q	0	0	0
	*selu*	Enterotoxin-like protein U	9^∗B^	0^∗B^	65^∗G,S^
	*egc*	Enterotoxin Gene Cluster (*seg/sei/selm/seln/selo/selu*)	9^∗B^	0^∗B^	65^∗G,S^
Other superantigens	*tst1* (“bovine” allele)	Toxic Shock Syndrome Toxin, allele from strain RF122	35^∗B^	40^∗B^	8^∗G,S^
	*etA/B/D*	Exfoliative toxins A, B, and D	0	0	0
	*pvl*	Panton Valentine Leukocidin	0	0	0
Resistance^1^	*tetK*	Tetracycline	12^∗B^	0	0^∗G^
	*fosB*	Metallothiol Transferase	38^∗S^	73^∗G^	19
Misc	*sdrD*	Sialoprotein-binding protein D	97^∗B^	100^∗B^	37^∗G,S^
	*splE*	Serine protease E	59^∗S,B^	27^∗G^	35^∗G^
	*lukM/lukF-PV* (P83)	Bovine leukocidin	21^∗S,B^	93^∗G^	72^∗G^
	*Q7A4X2*	Hypothetical protein	47^∗S,B^	80^∗G^	86^∗G^
	*ssl06/set21*	Staphylococcal superantigen-like protein 6	41^∗S,B^	73^∗G,B^	21^∗G,S^
	*ssl10/set4*	Staphylococcal superantigen-like protein 10	68^∗S^	93^∗G^	73

*A comprehensive list of the prevalence of all genes detected by DNA microarray is provided as a supplement. ^1^None of the isolates harbored the resistance genes *mecA, lnuA, msrA, mefA, mphC, vatA*/*B, vgaA/B, aacA-aphD, aadD, aphA3, sat, dfrS1, far1, Q6GD50, mupA, cat, fexA, cfr, vanA*/*Z, qacA*/*C.*^∗^The distribution of the respective gene differed significantly between strains from the stated sources (G, goat, S, sheep, B, bovine) with *p* ≤ 0.05.*

An overview of all CC and *spa* types detected is provided in **Table 2**. The isolates analyzed could be assigned to eleven different CC, of which only two (CC130 and CC133) were common among both caprine (71%) and ovine (93%) isolates. A total of 22 different *spa* types were detected. The most prevalent *spa* types were t1773 among the caprine and t1166 among the ovine *S. aureus* isolates, to which 26 and 27% of the analyzed isolates could be assigned, respectively. Three new *spa* types were detected: t15248, t15249, and t15404. While 51% of the bovine *S. aureus* isolates led to false-negative results in the Staphaurex latex agglutination test, all isolates from the milk of small ruminants tested in this study yielded positive results and were thus correctly identified as *S. aureus* by the Staphaurex latex agglutination test.

**Table 2:** Clonal complexes (CC) and *spa* types of the *S. aureus* isolates from goat and sheep milk.

Origin	CC	*n*	*spa* type (*n*)	Isolate ID
Goat (*n* = 34)	CC1	1	t127 (1)	G16
	CC5	1	t002 (1)	G25
	CC7	2	t091 (2)	G10, G29
	CC9	1	t899 (1)	G28
	CC30	1	t012 (1)	G09
	CC101	1	t056 (1)	G21
	CC130	15	t1773 (9)	G03, G05, G14, G17, G20, G22, G26, G27, G32
			t11826 (2)	G08, G15
			t15248 (4)	G06, G23, G31, G34
	CC133	9	t544 (1)	G18
			t1166 (2)	G12, G19
			t2678 (3)	G04, G07, G33
			t3583 (1)	G11
			t4735 (1)	G13
			t15249 (1)	G30
	CC398	1	t4475 (1)	G24
	CC522	2	t1534 (1)	G02
			t5428 (1)	G01
Sheep (*n* = 15)	CC97	1	t267 (1)	S05
	CC130	3	t1773 (1)	S09
			t11826 (1)	S14
			t15404 (1)	S06
	CC133	11	t998 (1)	S04
			t1166 (4)	S07, S08, S11, S15
			t2678 (2)	S10, S12
			t3583 (1)	S01
			t4735 (2)	S02, S03
			t6060 (1)	S13

An overview of the prevalence of the most important virulence and resistance genes detected by DNA microarray is provided in **Table 1**. The supplementary files include a comprehensive list of the prevalence rates of all genes detected (Supplementary Table S1) and a complete overview of all hybridization results (Supplementary Table S2). Overall, 67% of all isolates harbored at least one enterotoxin gene. The most prevalent enterotoxin genes were *sec* and *sel*, which were present in 55% of the isolates from small ruminants. The *sea* gene was found exclusively among caprine isolates. None of the genes encoding exfoliative toxins or Panton–Valentine leukocidin were detected. Virulence genes associated with the toxic shock syndrome were found in 27 isolates.

Seven isolates harbored genes conferring penicillin resistance (*blaZ/I/R*). Genes conferring tetracycline resistance were found only among the caprine isolates. All isolates harbored *sdrM*, which encodes a multidrug eﬄux pump. None of the caprine and ovine isolates harbored genes conferring resistance to methicillin, aminoglycosides, streptogramin A, virginiamycin A, glycopeptides, and vancomycin.

## Discussion

The prevalence of *S. aureus* in caprine and ovine bulk tank milk samples varies depending of the country. Muehlherr et al. (2003) detected *S. aureus* in 32% of the caprine and 33% of ovine bulk tank milk samples in Switzerland, while Linage et al. (2012) and Álvarez-Suárez et al. (2015) detected coagulase positive staphylococci in 66% of caprine and 15% of ovine bulk tank milk samples in Spain. Considering the very low prevalence of *S. aureus* detected among the analyzed milk samples of single animals in this study, the overall detected prevalence of *S. aureus* in the bulk milk samples examined was high. This suggests that the prevalence of *S. aureus* as a subclinical agent of mastitis in small ruminant herds in Switzerland may have been underestimated. This is of particular relevance, as SFP has been associated with raw milk from small ruminants (Giezendanner et al., 2009) and as traditional goat and sheep raw milk cheeses are popular.

Most of the isolates characterized in this study were assigned to CC130 and CC133, suggesting that these lineages may represent the major CC among caprine and ovine *S. aureus* isolates in Switzerland. These results are consistent with the findings of previous studies suggesting that predominance of either CC130/CC133 or CC522 in *S. aureus* isolated from milk of small ruminants is associated with geographical, breed- and infection-related aspects (Concepción Porrero et al., 2012; Eriksson et al., 2013; Shepheard et al., 2013; Smith et al., 2014). Only few CC (CC7, CC9, CC97) were detected among strains of both small ruminants and cows.

Even though *S. aureus* isolates originating from caprine and ovine hosts have been *spa* typed in several recent studies (Concepción Porrero et al., 2012; Eriksson et al., 2013; Smith et al., 2014; Bar-Gal et al., 2015), three new *spa* types were detected among the isolates in this study. This suggests that to date, data on the population structure of *S. aureus* isolates originating from small ruminants is still very limited. The *agr* types and *cap* genes detected in this study are consistent with the findings of previous studies investigating *S. aureus* from small ruminants (Alves et al., 2009; Vautor et al., 2009; Bar-Gal et al., 2015).

Most of the isolates analyzed from small ruminants in this study were lacking antibiotic resistance genes. Resistance gene profiles from caprine and ovine strains in this study were not significantly different from those of bovine isolates (Moser et al., 2013). Only the presence of *tetK* in 12% of the caprine isolates was significantly higher compared to ovine and bovine isolates (*p* = 0.007). Overall, the prevalence of *blaZ/I/R* (14%), *tetK* (8%), *tetM* (2%), *ermA/B/C* (2%) detected was lower than the prevalence detected when analyzing *S. aureus* from small ruminants milk or nasal swabs in recent studies from the Middle-East and Africa (Gharsa et al., 2012; Bar-Gal et al., 2015; Jamali et al., 2015). The prevalence of antibiotic resistance genes detected was surprisingly high, considering that herd management differs vastly in small ruminants and cattle, with culling being preferred to antimicrobial treatment in small ruminants.

All isolates harboring *tst1* also harbored the genes *sec* and *sel*, and were assigned exclusively to CC130 and CC133. These genes are located on the ovine pathogenicity island *SaPIov1* (Guinane et al., 2010), and have been previously reported in isolates originating from small ruminants (Smyth et al., 2005; Gharsa et al., 2012). Consistent with findings among *S. aureus* from sheep and goats in Israel (Bar-Gal et al., 2015), in this study, the prevalence of *tst1, sec*, and *sel* was significantly higher among small ruminant isolates than among bovine isolates (*p* < 0.003), which in contrast are more likely to harbor *egc* genes (*p* = 0.000). In this study, the detected overall prevalence of 67% of *S. aureus* carrying at least one enterotoxin gene was similar to 65% reported by Scherrer et al. (2004).

Many genes encoding virulence factors were present at similar rates in caprine, ovine and bovine isolates. This included genes encoding hemolysins (*hla, hlb, hld*), adhesion factors (*clfA, clfB, ebps, fib, fnbA, vwb*), hyaluronate lyase (*hysA1/A2)*, immunodominant antigen B (*isaB*), transferrin binding protein (*isdA*) and serine proteases (*splA, sspa*). In several studies, these virulence factors have been reported to play a role in mastitis in cattle (Viana et al., 2010; Ote et al., 2011; Wolf et al., 2011). While many genes were equally distributed among small ruminant and bovine isolates, statistically higher prevalence rates of *cap8, sdrD, sec, sel, tst1, ssl06, edinB*, and *Imrp* (*RF122*) among *S. aureus* from small ruminants were observed. As for genes associated with biofilm formation (*icaA/C/D*), very high prevalence rates have been previously reported in isolates originating from small ruminants in particular (Bar-Gal et al., 2015) and from ruminants in general (Snel et al., 2014; Prenafeta et al., 2014).

Comparison of goat and sheep isolates tested in this study showed that caprine and ovine *S. aureus* exhibited highly similar virulence and resistance gene patterns. However, some species-specific patterns were observed. Higher prevalence rates of *splE* among the caprine (*p* = 0.038) and of *lukM* (*p* = 0.000) among the ovine isolates was observed. Simultaneous presence of *splE* and *sdrD*, which was detected in four ovine and 19 caprine isolates in this study, has been associated with gangrenous mastitis in small ruminants (Vautor et al., 2009). In contrast, *lukM* was associated with high leukotoxic activity against bovine polymorphonuclear leukocytes (Rainard et al., 2003) and was hypothesized to play a central role in mastitis in ruminants (Barrio et al., 2006). In addition, significant differences in the prevalence of genes *ssl06/set21, ssl10/set4*, and *Q7A4X2* in caprine compared to ovine isolates were observed. While genes encoding for superantigen-like proteins (*ssl*), have been associated with immunoevasion by interfering with the toll-like receptor system (Zecconi and Scali, 2013), Q7A4X2 may be involved in biofilm formation (Snel et al., 2014). These findings suggest that the virulence genes detected, and especially *lukM, sdrD*, and *splE*, represent important virulence factors for *S. aureus* strains causing mastitis in small ruminants.

Finally, the performance of the Staphaurex latex agglutination test for identification of *S. aureus* from small ruminants was assessed, as this test was reported to yield false-negative results in 51% of all bovine *S. aureus* strains tested (Moser et al., 2013). The results of this study show that the Staphaurex latex agglutination test system is a highly reliable diagnostic tool for identification of *S. aureus* isolates from caprine and ovine milk samples.

## Conclusion

The findings of this study suggest that *S. aureus* shows pronounced adaptation to small ruminants in general, but not to sheep or goats in particular. Comparing *S. aureus* from caprine, ovine and bovine milk samples collected in the same country, some common virulence genes were observed, but the results indicate that *S. aureus* from small ruminants may form a distinct population. Further studies covering an extensive strain collection of *S. aureus* from small ruminants collected at various geographical locations are needed to ensure that this finding can be extrapolated to *S. aureus* in general. Although low prevalence rates of *S. aureus* on the level of single animals exhibiting signs of mastitis was detected, 46% of the bulk tank milk samples from small ruminants were positive for *S. aureus.* This suggests that *S. aureus* may pose problems for animal and consumer health, in particular, as many products made from the milk of small ruminants are consumed raw.

## Author Contributions

SJ and RS conceived and designed the study. AM carried out the laboratory work. AM and SJ analyzed and interpreted the data. AM and SJ wrote the manuscript. All authors critically revised and approved the final manuscript.

## Conflict of Interest Statement

The authors declare that the research was conducted in the absence of any commercial or financial relationships that could be construed as a potential conflict of interest.

## Footnotes

1 http://www.splitstree.org/

2 http://www. spaserver.ridom.de/
